# Supplementary material for: Glycosylation Modulation Dictates Trafficking and Interaction of SARS-CoV-2 S1 Subunit and ACE2 in Intestinal Epithelial Caco-2 Cells
Source: Biomolecules. 2024 Apr 30;14(5):537. doi: 10.3390/biom14050537 (PMC11117975; doi:10.3390/biom14050537)
Supplement: Supplementary file 1 [file biomolecules-14-00537-s001.zip › biomolecules-2990524-supplementary-final.pdf]

Glycosylation Modulation Dictates Trafficking and Interaction of SARS-CoV-2 S1 Subunit and ACE2 in Intestinal Epithelial Caco-2 Cells

Marianne El Khoury, Dalanda Wanes, Maura Lynch-Miller, Abdullah Hoter, Hassan Y. Naim*

Supplementary Materials

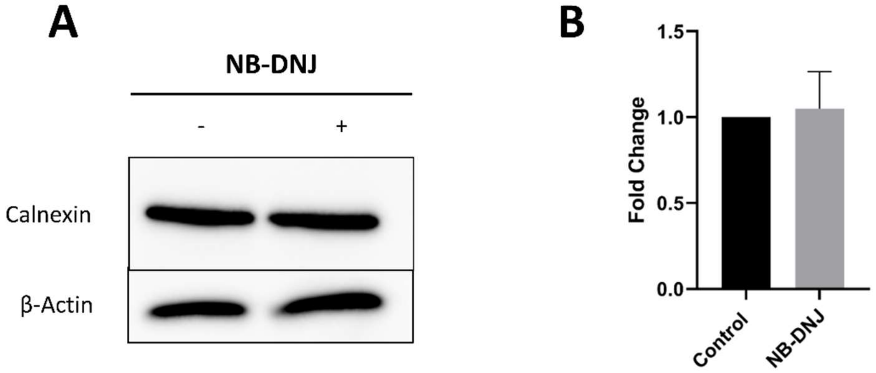

Supplementary Figure S1 NB-DNJ treatment of COS-1 cells. (A) COS-1 cells were treated with NB-DNJ for 24 hours. The cells were lysed post-treatment and an equal amount of proteins was analyzed by Western blot. **(B)** Analysis of the results obtained in A. The values represented are normalized to β-actin. Unpaired t-test, versus non-treated, S.E.M., *n* = 3.

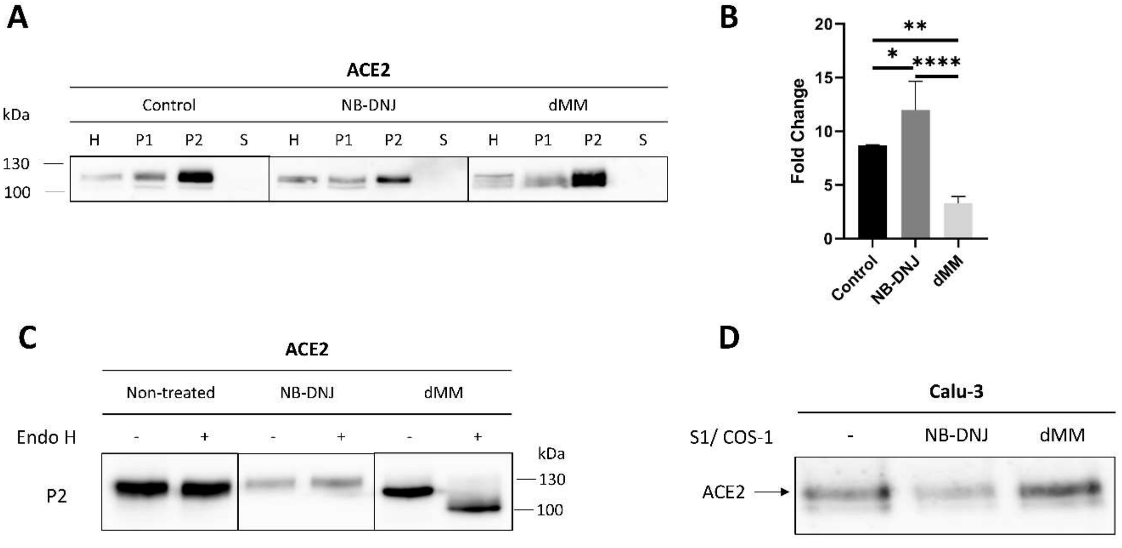

Supplementary Figure S2. Effect of NB-DNJ and dMM on the Trafficking of and Interaction with ACE2 in Calu-3 cells. (A) Calu-3 cells were treated with NB-DNJ and dMM for 48 hours. Total homogenates (H) were

fractionated into intracellular and basolateral membranes (P1), apical membrane/BBM fraction (P2) and a fraction containing cytosolic vesicles (S). The fractions were then analyzed by Western blot. **(B)** Analysis of the results obtained in A by normalizing the P2 fraction to H (P2/H). **(C)** ACE2 from the apical membranes of control and treated cells were treated with endo-H. The blots were taken from different experiments. **(D)** Calu-3 cells were used to validate the functionality of the *in vitro* system used for interaction studies in intestinal epithelial cells. Tukey's multiple comparisons test, ** $p < 0.01$, versus Control P2, S.E.M., $n = 3$.
