# Supplementary material for: Glycosylation Modulation Dictates Trafficking and Interaction of SARS-CoV-2 S1 Subunit and ACE2 in Intestinal Epithelial Caco-2 Cells
Source: Biomolecules. 2024 Apr 30;14(5):537. doi: 10.3390/biom14050537 (PMC11117975; doi:10.3390/biom14050537)
Supplement: Supplementary file 1 [file biomolecules-14-00537-s001.zip › biomolecules-2990524-WB original.pdf]

Original Uncropped Blots

Merged with Marker

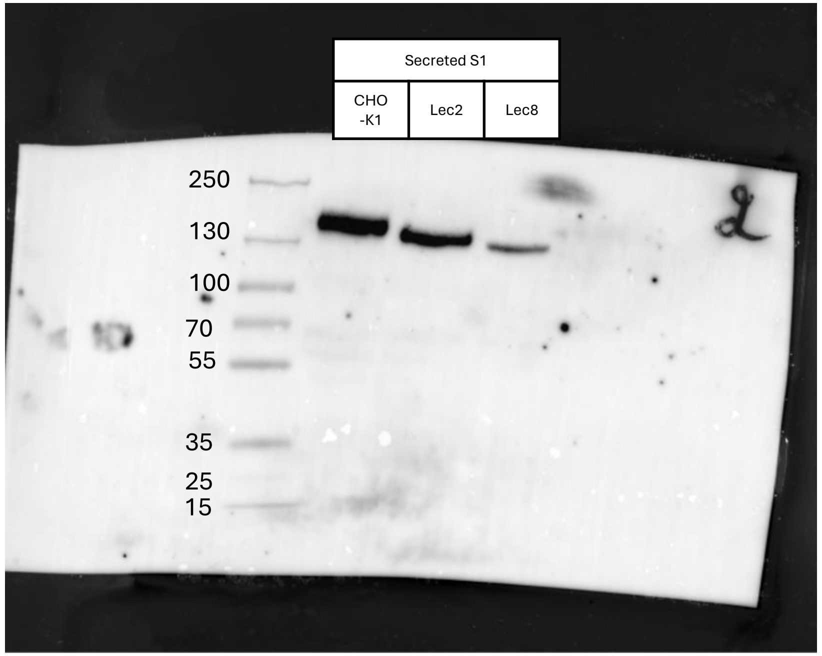

Fig S1A

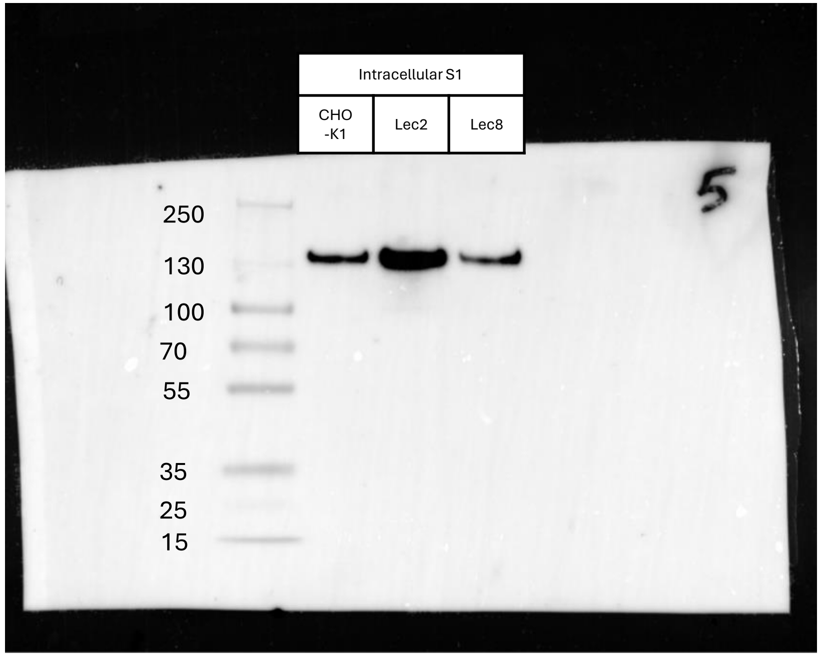

Fig S1C

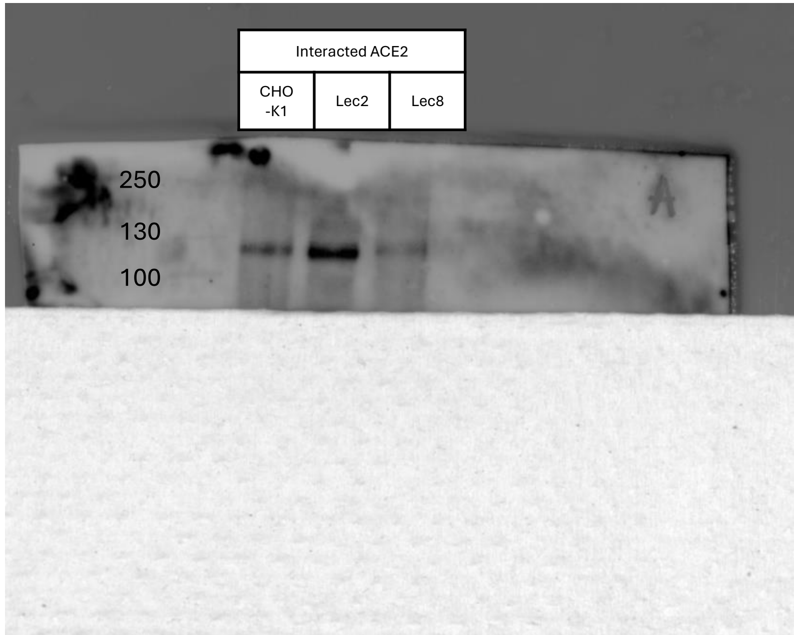

Fig S2A

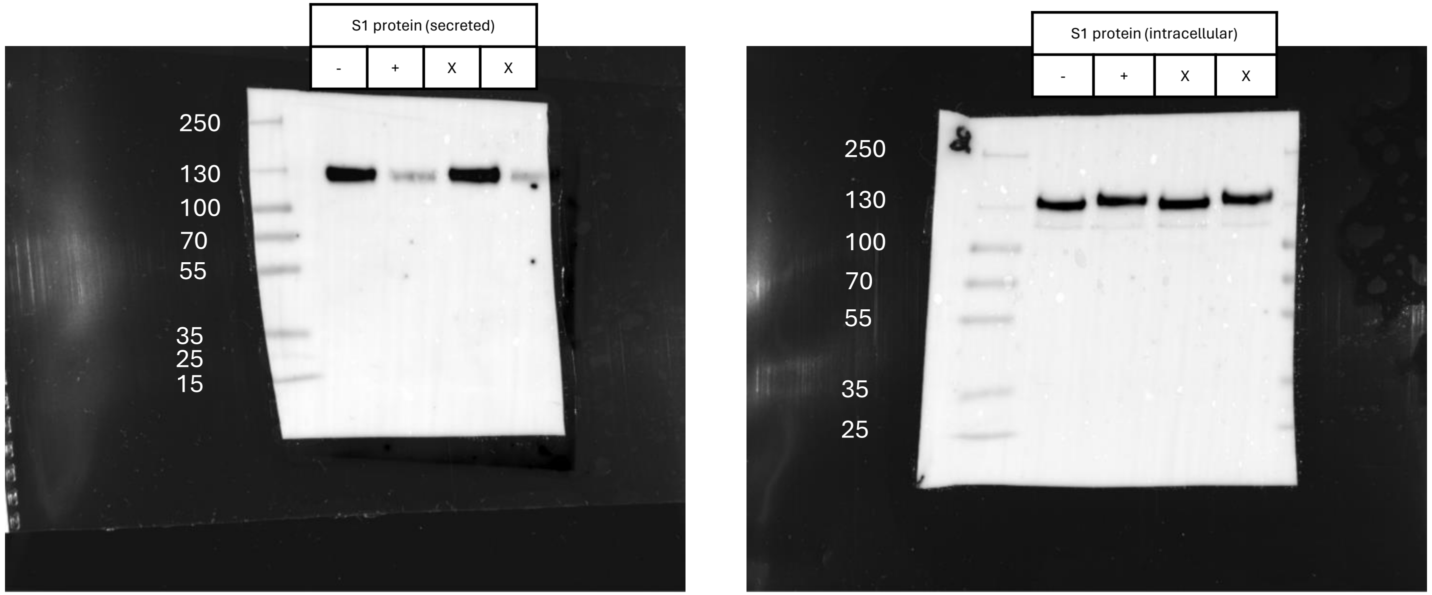

Fig S3A

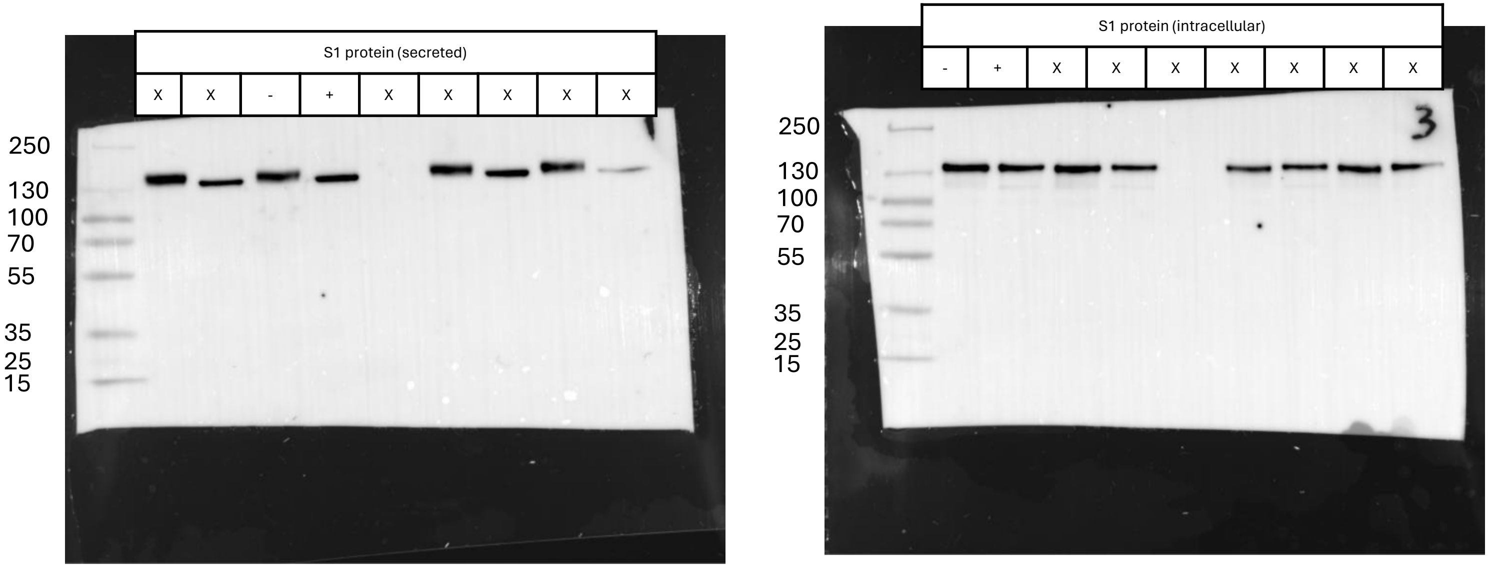

Fig S3C

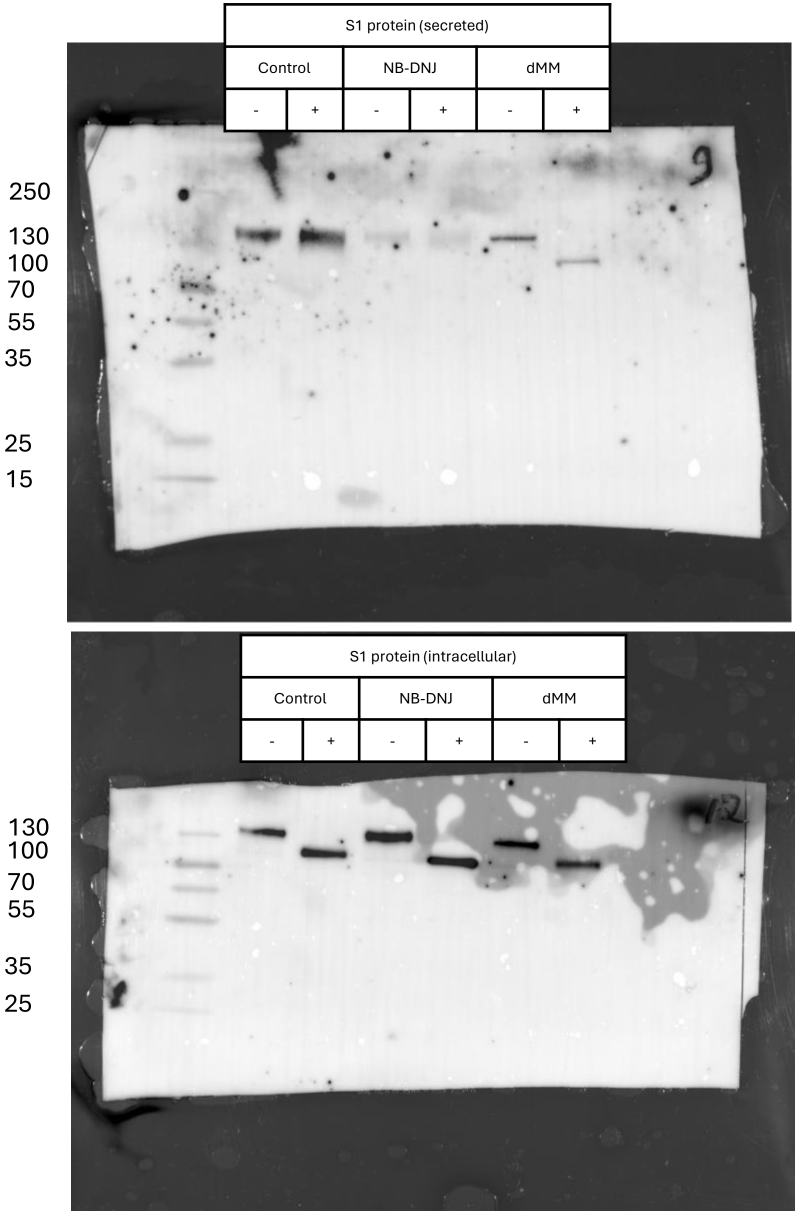

Fig S3G

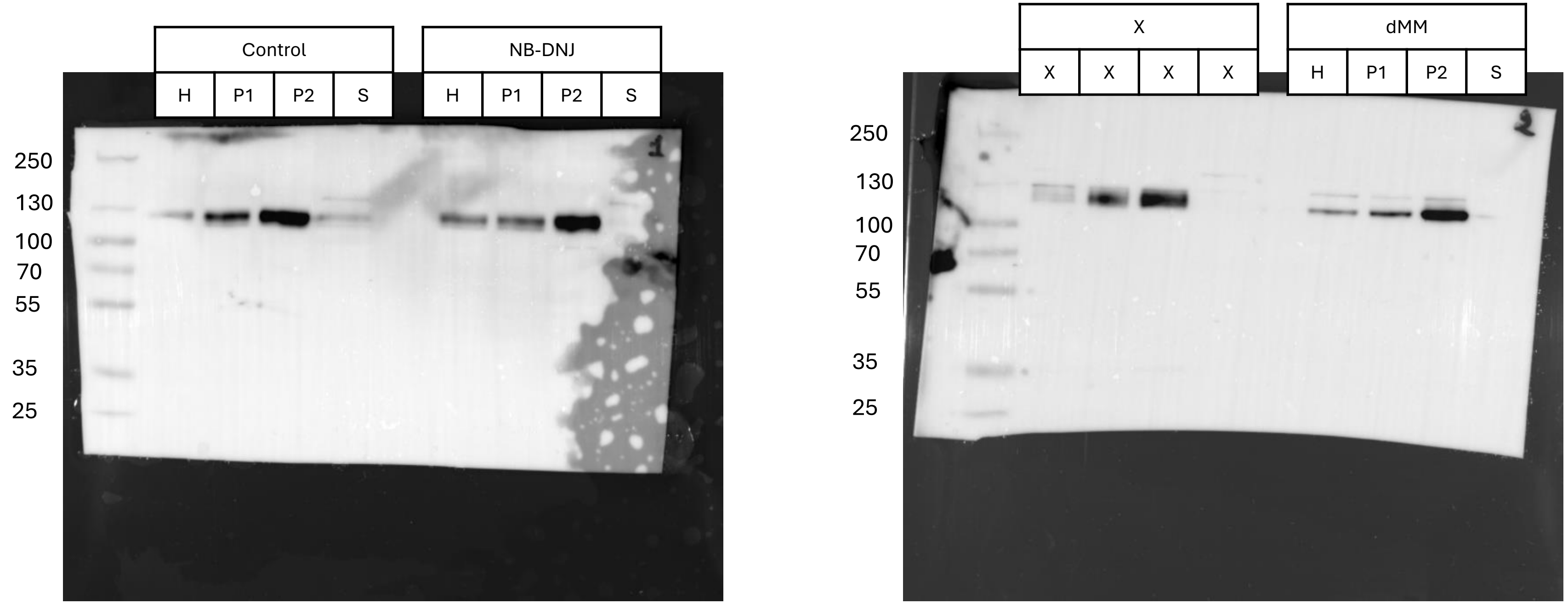

Fig S4A

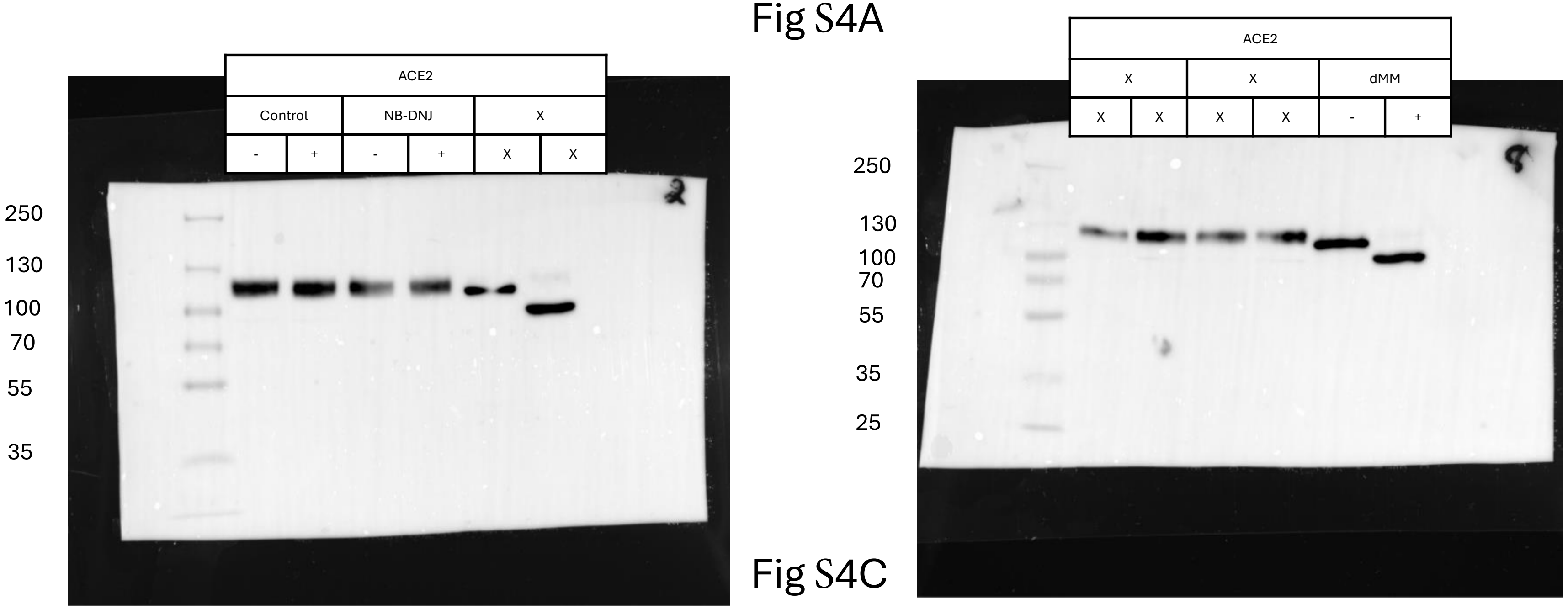

Fig S4C

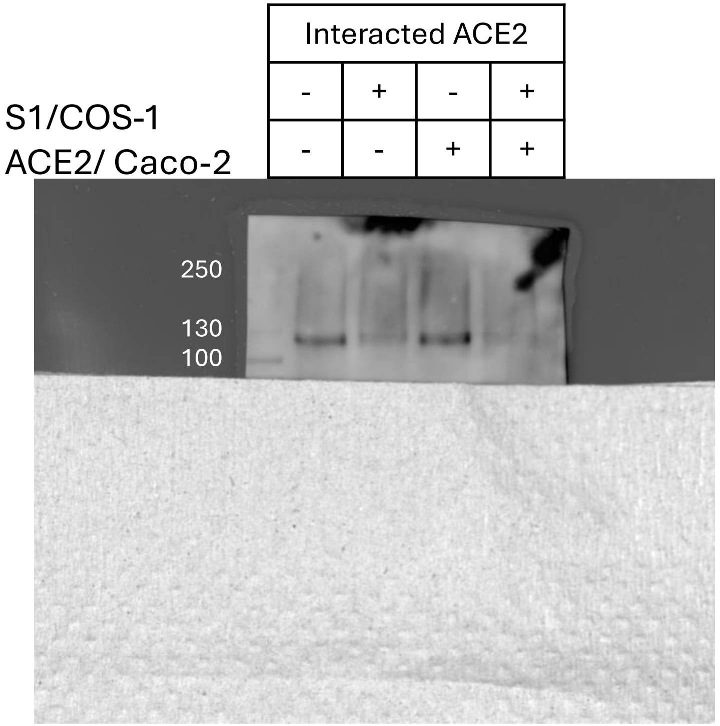

Fig S5A

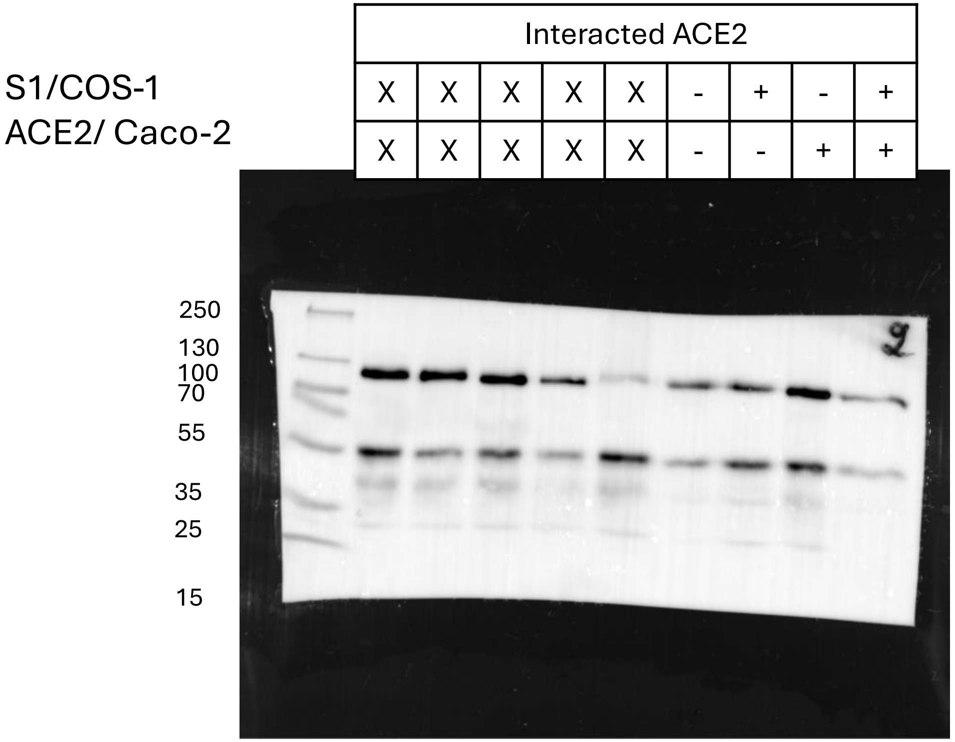

Fig S5C

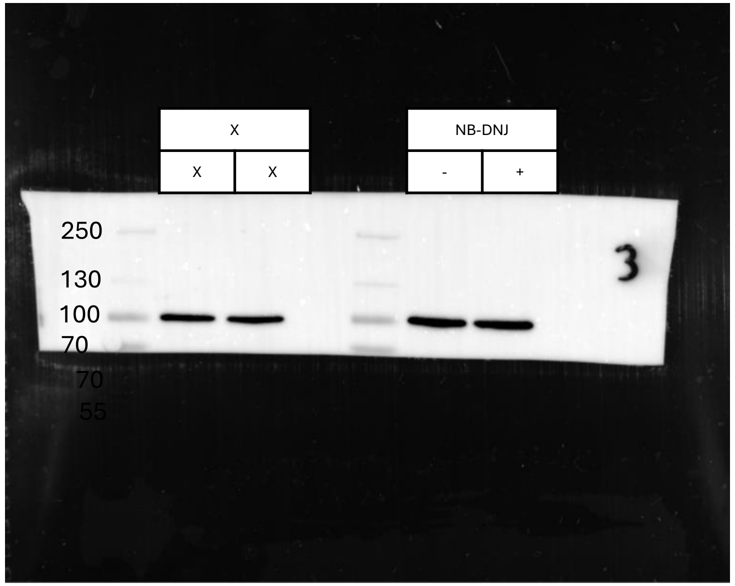

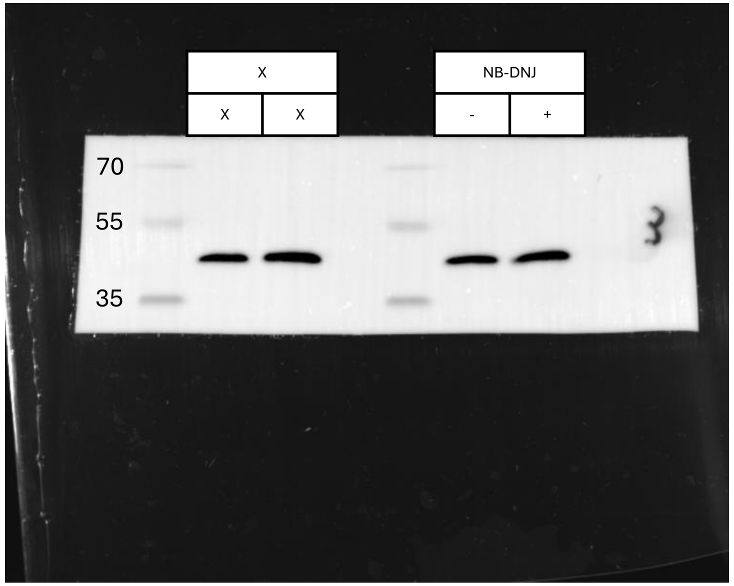

Supplementary Figure S1

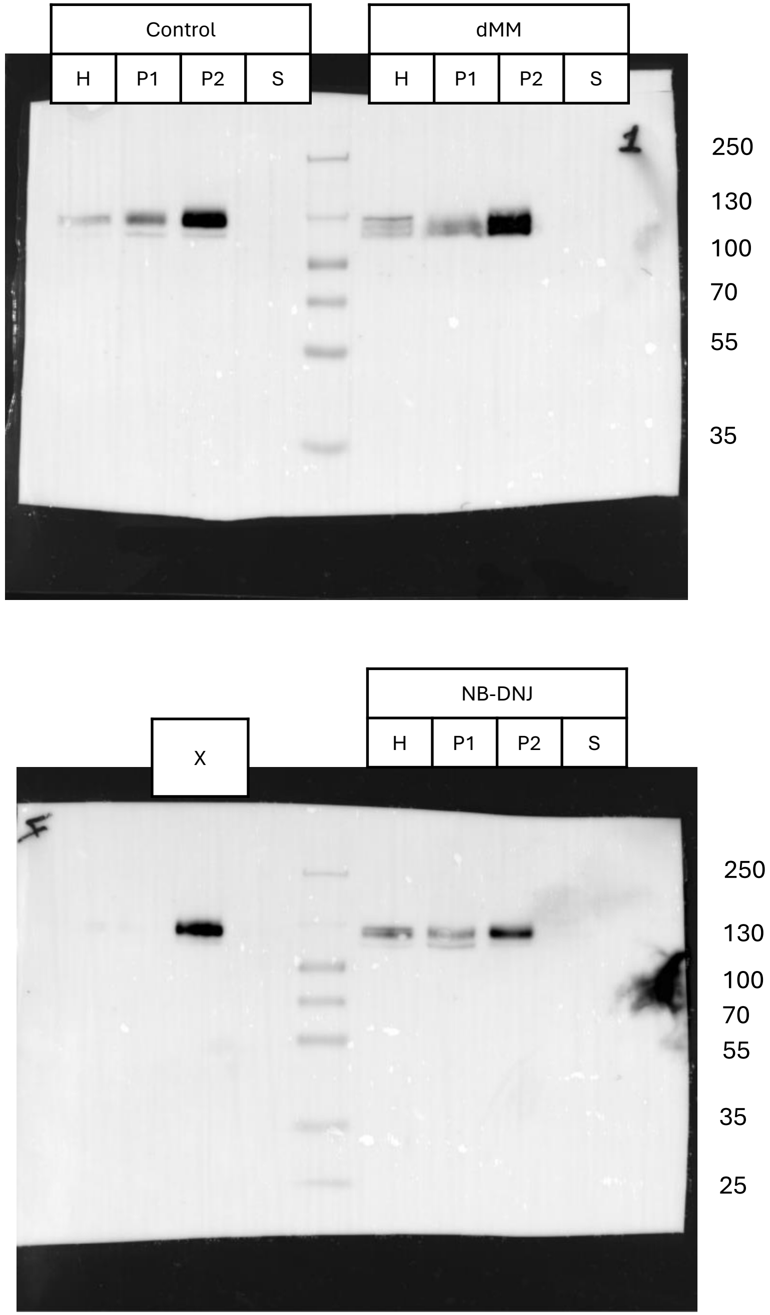

Supplementary Figure S2A

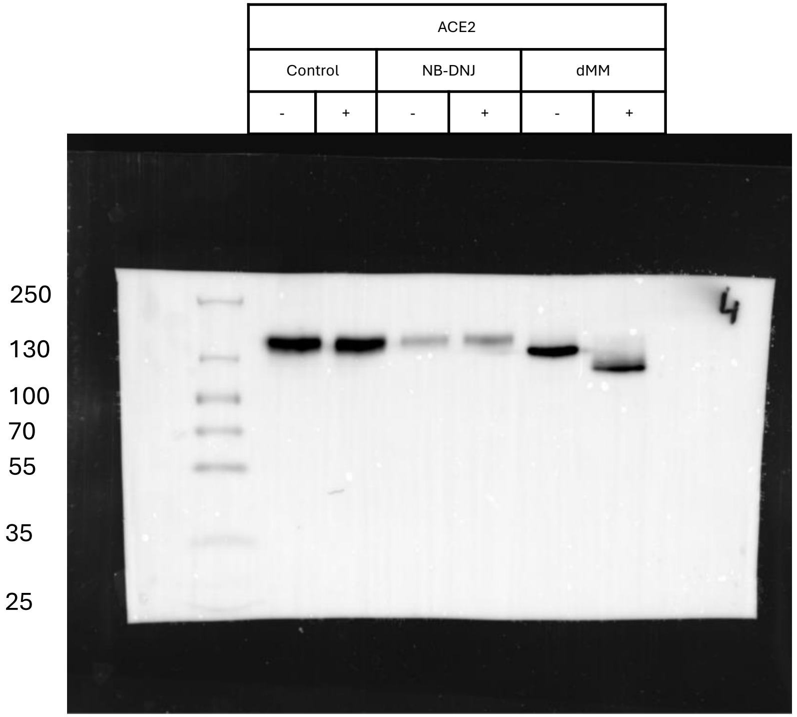

Supplementary Figure S2C

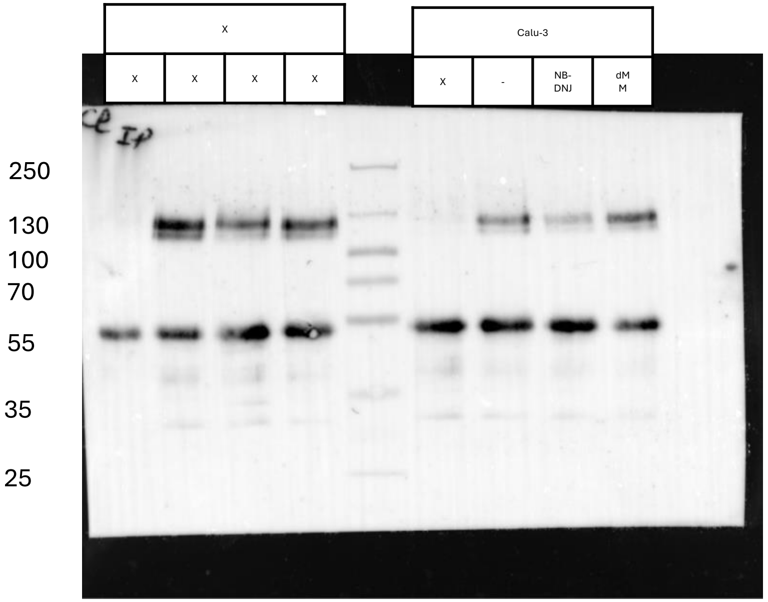

Supplementary Figure S2D
